# P-1924. Antifungal Resistance of Candida spp. isolates from HIV/AIDS Patients in Iran 1999-2024: A Systematic Review and Meta-analysis

**DOI:** 10.1093/ofid/ofaf695.2093

**Published:** 2026-01-11

**Authors:** Aliasghar Fakhri-Demeshghieh, Ali Yousefipour, Yousef Bolouki, Mohammad Sadegh Bigdeli, Erfan Zamani, Amirreza Farajkhoda, Alireza Salmani, Farzad Katiraee, Saied Bokaie

**Affiliations:** DVM, Specialty board-certified in epidemiology, Department of Food Hygiene and Quality Control, Epidemiology & Zoonoses Division of Faculty of Veterinary Medicine, University of Tehran, Tehran, Iran. https://orcid.org/0000-0003-1693-533X., Tehran, Tehran, Iran; Department of Pathobiology, Faculty of Veterinary Medicine, University of Tabriz, Tabriz, Iran., Tabriz, Azarbayjan-e Sharqi, Iran; Department of Pathobiology, Faculty of Veterinary Medicine, University of Tabriz, Tabriz, Iran., Tabriz, Azarbayjan-e Sharqi, Iran; Department of Pathobiology, Faculty of Veterinary Medicine, University of Tabriz, Tabriz, Iran., Tabriz, Azarbayjan-e Sharqi, Iran; Department of Pathobiology, Faculty of Veterinary Medicine, University of Tabriz, Tabriz, Iran., Tabriz, Azarbayjan-e Sharqi, Iran; Department of Pathobiology, Faculty of Veterinary Medicine, University of Tabriz, Tabriz, Iran., Tabriz, Azarbayjan-e Sharqi, Iran; Department of Pathobiology, Faculty of Veterinary Medicine, University of Tabriz, Tabriz, Iran., Tabriz, Azarbayjan-e Sharqi, Iran; Associate professor, Section of Mycology, Department of Pathobiology, Faculty of Veterinary Medecine, University of Tabriz, Tabriz, Iran. https://orcid.org/0000-0002-8634-4378., Tabriz, Azarbayjan-e Sharqi, Iran; Professor of Epidemiology, Department of Food Hygiene and Quality Control, Epidemiology & Zoonoses Division of Faculty of Veterinary Medicine, University of Tehran, Tehran, Iran., Tehran, Tehran, Iran

## Abstract

**Background:**

Antifungal resistance in Candida species in individuals living with HIV/AIDS infections due to the scarcity of antifungal agents is important especially in Iran with an estimate of 46000 individuals living with HIV. This systematic review and meta-analysis aimed to determine the resistance rate of Candida spp. isolates from HIV/AIDS positive individuals in Iran 1999-2024.

**Methods:**

PubMed, Scopus, Web of Science, MagIran, Scientific Information Database, and Google Scholar were searched. For each database, title-and-abstracts screening was independently performed by two review authors. The assessment of the risk of the bias of the included studies and the data extraction were performed independently by two review authors using JBI Critical Appraisal Checklist for Studies Reporting Prevalence data. Wherever suitable, meta-analysis was performed using a random-effects model with Freeman-Tukey double arcsine transformation.

**Results:**

From the retrieved 4925 references, 18 references related to 15 studies with 1318 Candida spp. isolates were included. The highest pooled resistance rate of Candida spp. was observed against clotrimazole (21%, 95% CI: 14-30, I^2^=67.29%) while the lowest pooled resistance rates of Candida spp. were observed against voriconazole, caspofungin, amphotericin B, and nystatin with the pooled estimates of zero percent. In species level, the highest pooled resistance rates were observed for Candida krusei against fluconazole (72%, 95% CI: 0-100%) and itraconazole (38%, 95% CI: 17-62%) and for Candida glabrata against clotrimazole (38%, 95% CI: 27-50%).

**Conclusion:**

The use of amphotericin B, caspofungin, and nystatin for the treatment of Candida spp. in HIV-AIDS patients in Iran is recommended based on clinical indication.

**Disclosures:**

All Authors: No reported disclosures

